# Assessment of the Substance Antioxidative Profile by Hyaluronan, Cu(II) and Ascorbate

**DOI:** 10.3390/pharmaceutics13111815

**Published:** 2021-10-31

**Authors:** Katarína Valachová, Ladislav Šoltés

**Affiliations:** Centre of Experimental Medicine, Institute of Experimental Pharmacology and Toxicology, Slovak Academy of Sciences, Dúbravská Cesta 9, SK-84104 Bratislava, Slovakia; ladislav.soltes@savba.sk

**Keywords:** preventive antioxidants, chain-breaking antioxidants, reactive oxygen species

## Abstract

In the minireview presented here, the authors discuss the evaluation of inhibitory effect of substances in the phases of initiation and propagation of high-molar-mass hyaluronan oxidative degradation. The experimental approach should be considered as original since on using a simple experimental assay it is possible to prove both the so-called “preventive” and “chain-breaking” antioxidant activity of investigated water-soluble endo- or exogenous substances.

## 1. Introductory Remarks

The website “Free Radical School Presentations” [1] serves as “an archive” for many of the Free Radical School Workshop and Virtual School lectures that were organized by the Society for Redox Biology and Medicine and presented at SfRBM’s Annual Meeting within 15 years (the first one in 1997 and the last one in 2011). This website is intended to be a source for basic education and reference material for a wide variety of important topics in the field of redox research including the experimental methods to assess the substance antioxidative profile.

In the 1990s, the research activities of many scientific teams directly or indirectly focused on the thesis that antioxidants are the “miracle substances” that can effectively intervene in both the acute and chronic phases of many diseases, including aging. Reaction systems for evaluating the antioxidant activity of a substance also contain a generator of oxidant(s) and a probe whose intactness during the reaction proves the antioxidant activity of the substance assessed.

In this minireview the primary/initiating oxidants are the OH radicals, the secondary oxidants are alkyloxy- and alkylperoxy- type radicals. The probe is the organism’s own macromolecule, namely a high-molar-mass hyaluronan. The kinetics of the hyaluronan oxidative degradation has been most properly monitored by changing the dynamic viscosity of the reaction solution.

## 2. Sequestration of Copper Cations with Ascorbate

The biogenic transition metal—copper—is found in the healthy human body as cupric and cuprous cations, whereas 95% of the total copper content is bound to the blood plasma protein—ceruloplasmin [2,3,4]. Each ceruloplasmin macromolecule binds eight copper cations, of which two are readily released. The normal level of serum ceruloplasmin concentration is in a range of 1.52–2.65 µmol/L. Thus, under physiological conditions the concentration of loosely bound copper (cupric/cuprous cations) can reach up to ≈0.66 μmol/L. The patho-physiological range of ceruloplasmin blood serum level is significantly increased, since this metalloprotein belongs to the acute phase reactants, the level of which rises during acute and chronic inflammations, infection, trauma, Alzheimer’s disease, etc. Ascorbic acid/vitamin C, in the form of ascorbate, is ubiquitous in the human body. The ascorbate molecule (Asc^−^) in a living organism functions also as a coordinating low molar mass ligand. In case of ascorbate-iron pair, ascorbate may serve both as a Fe(III)/Fe(II) chelating agent and reductant of Fe(III) to Fe(II) cations. In latter case, under the aerobic conditions ascorbate with ferrous cation forms a coordination complex: ascorbate---Fe(II)---dioxygen [5]. This, the so called Udenfriend oxidative complex/system, is a very efficient oxidative agent used, e.g., by organic chemists to hydroxylate aromatic compounds, saturate hydrocarbons to alcohols, olefins to epoxides, etc. [6].

**Scheme 1:**
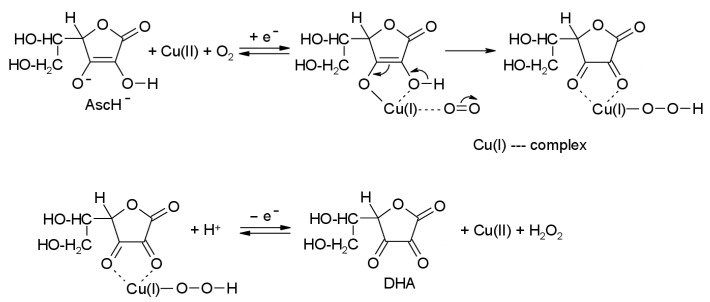
Generation of H_2_O_2_ via the Weissberger system composed of ascorbate and Cu(II) under aerobic conditions. AscH^−^ represents ascorbate anion and DHA represents dehydroascorbate.

The paramagnetic bivalent Cu(II) (outermost orbitals = 3d^9^) represents the most stable oxidation state of copper. Since ascorbate acts as a powerful reducing agent with a standard reduction potential E’° of +0.282 V for the redox couple Asc^•−^/Asc^−^ at pH 7, it should reduce Cu(II) to Cu(I). Thus, taking into account that the standard reduction potential of the pair Cu(II)/Cu(I) is +0.16 V, Cu(I) should be able to reduce O_2_ molecules to yield directly H_2_O_2_. However, as often claimed, the so called Weissberger system—ascorbate---Cu(II/Cu(I)---dioxygen [7,8,9]—generates hydrogen peroxide molecules (cf. Scheme 1) [10,11,12,13], or due to the decomposition of H_2_O_2_ by Cu(I) complex through Fenton type reaction, the Weissberger system became one of the most potent generators of hydroxyl radicals [14,15,16].

When the Weissberger system has no additional oxidizable substrate, we speak about ascorbate autoxidation [17]. In any case, it should be pointed out that the biological consequences of interactions of vitamin C with biogenic transition metal cations of iron, copper, or manganese have not been fully understood yet. Over the past decade, the pro-oxidant properties of ascorbate have been investigated in addition to its better explored antioxidant role.

When oxidizable substrates are simultaneously present, the reaction products consist of both oxidized/decomposed substrate [18] and DHA—dehydroascorbate, whose molecules in aqueous milieus hydrolyze fast to 2,3-diketo-l-gulonic acid [17]. Although for the next sections of this minireview the complexation of ascorbate with another biogenic transition metal is not essential, the reader could find some complexation kinetics and/or equilibrium data between ascorbate and several metal cations, e.g., in the paper by Fornaro and Coichev [19]. One of the frequently cited statements in literature is that copper cations released from the ceruloplasmin macromolecule are extensively entrapped by albumin present in the bloodstream. This tenet is naturally in part true, but it must also be admitted that the anions of ascorbate are the most probably co-responsible for the release of copper cations from ceruloplasmin. The charge–charge interaction between AscH^−^ and, e.g., [CuCl]^+^ with a rate constant of 280 mol^−1^ L^−1^ s^−1^ [19] may outweigh the rate of gradual dissociation of the complex between [AscH]^−^ and [CuCl]^+^ followed by a subsequent association of Cu(II) with a copper fixing/binding site on the albumin molecule.)

## 3. Hyaluronan—Oxidizable Biological Substrate

**Figure 1:**
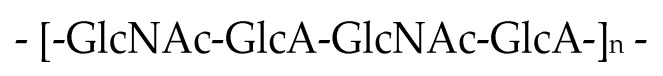
Hyaluronic acid.

Hyaluronic acid (hyaluronan; HA; Figure 1), or its salts, is a linear high-molar-mass natural polysaccharide formed from disaccharide units of regularly alternating *N*-acetyl-d-glucosamine (GlcNAc) and d-glucuronic acid (GlcA) units linked by β-(1→3) and β-(1→4) linkages. The chemical structure of HA is regular, the only exception is a possible replacement of *N*-acetyl-D-glucosamine by deacetylated glucosamine residues.

A human body weighing about 70 kg contains approx. 15 g of HA [20], whereas one third of this amount is turned over every day. Such an unusually extensive de novo synthesis of HA megadalton macromolecules suggests that the functions of both native hyaluronan and its lower sized fragments in the body will be diverse [21]: In the body of vertebrates HA is abundantly present in almost all body fluids and tissues. In a fibrous tissue a capsule called synovium, one of the main components is the synovial fluid (SF), functions as a lubricant [22,23,24,25,26,27,28]. The SF in a healthy human being, along with the blood plasma filtrate, also contains the entangled macromolecules of HA {1.4–3.6 mg/mL [26]}. While in SF and vitreous humor HA macromolecules are not associated with proteins, the HA chains, filling the space between the collagen fibrils, provide elastic properties of these soft tissues [29,30,31]. Yet, HA in extracellular matrices is linked with several proteoglycans creating a scaffold in the forms of more or less hard tissues such as skin, umbilical cord, and cartilage [32,33,34,35,36].

Two ways of HA decay must be denoted here: first, enzymatic depolymerization, by which HA fragments of lower molar mass are formed, and second, oxidative degradation, a process which is of great interest to polymer chemists, biochemists and molecular biologists [37,38,39,40,41].

The half-life of HA depolymerization (by hyaluronidases) is in the range of 1–3 weeks in cartilage, 1–2 days in skin and only 2–5 min in blood plasma [42,43]. In contrast, in SF of healthy individuals, whose fluid lacks any hyaluronidases, the half-life of HA ≈ 12 h is suggestive of other than enzymatic decay [44]. Such a rapid turnover of HA in SF could be elucidated by a mechanical pumping-out of a part of SF through the lymphatic system during the day-time moving activity of a person. Another proposed mechanism is an oxidative degradation of several megadalton-HAs in SF of healthy individuals [45,46,47,48]. This tenet is supported indirectly by the fact that under physiological conditions, the concentration of ascorbate in SF of healthy humans reaches the values closely to those in blood plasma, i.e., 40–140 μmol/L [49,50].

**Figure 2:**
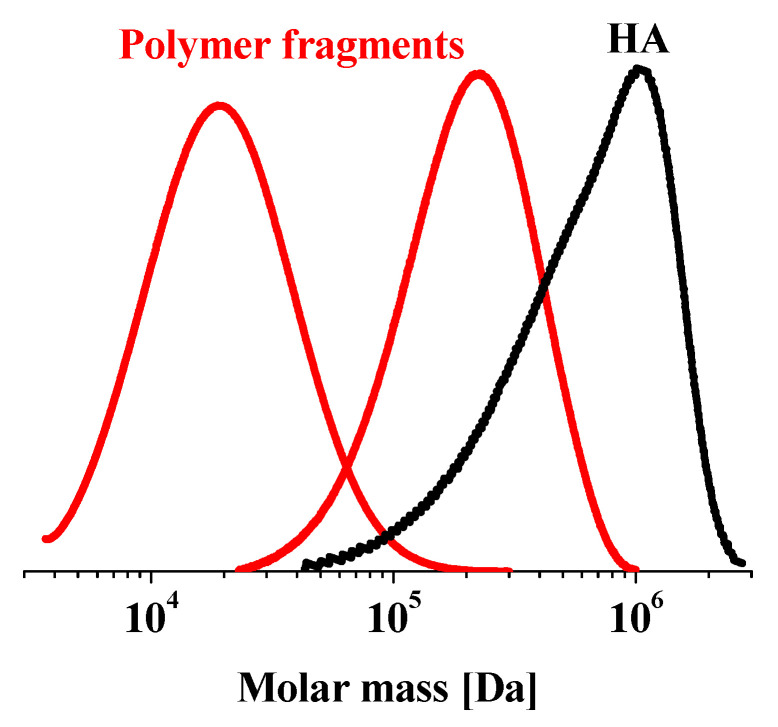
Comparison of the molar mass distribution of the megadalton HA sample and those ones degraded in situ under aerobic condition by action of AscH^−^ and copper ions. Adapted from [51], published by Elsevier, 2007.

Figure 2 illustrates the action of oxidative system comprising complex of ascorbate---Cu(II/Cu(I)---dioxygen: As evident, a megadalton-HA sample progressively degrades to intermediate-sized polymer fragments, which mean molar mass is reduced by one or even two orders of magnitude.

### 3.1. Hyaluronan Oxidative Degradation by Free-Radical-Chain Reaction

#### 3.1.1. Initiation Reaction(s)

As shown in the sub-section “Sequestration of copper cations with ascorbate” for the generation of ^•^OH radical(s) the so called Weissberger biogenic oxidative system can produce a continual flux of hydroxyl radicals [15,16]. (To simplify some reaction sequences appearing in this minireview, HA abbreviates the hyaluronan macromolecule. The A^•^ denotes a *C*-centered hyaluronan macroradical, a peroxy-type macroradical AOO^•^ along with a highly unstable alkoxy-type macroradical AO^•^ represents *O*-centered intermediate macroradicals).

**Scheme 2:**
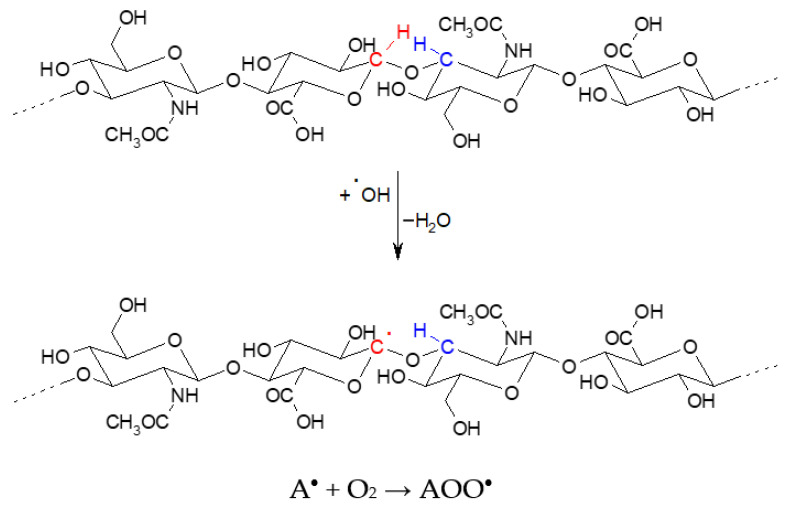
An intact HA macromolecule (upper panel) reacts with ^•^OH radical yielding an intermediate A^•^, i.e., a *C*-centered hyaluronan macroradical (lower panel), which immediately reacts with a molecule of oxygen resulting in a peroxy-type macroradical AOO^•^.

The following Scheme 2 describes structural chemical formulae the phase of initiation reaction(s) of high-molar-mass HA oxidative degradation:

The macroradical AOO^•^ can undergo the reaction with Cu(I) complex (cf. Scheme 1) yielding a highly unstable alkoxy-type macroradical. It is comprehensible that either AOO^•^ macroradical or that of AO^•^, along with the parent A^•^ intermediate macroradical, form a collection of highly reactive compounds, i.e., the initiators of subsequent self-perpetuating free-radical degradation of high-molar-mass HA.

#### 3.1.2. Transfer of the Free-Radical Centre and Fragmentation Reaction(s)

**Scheme 3:**
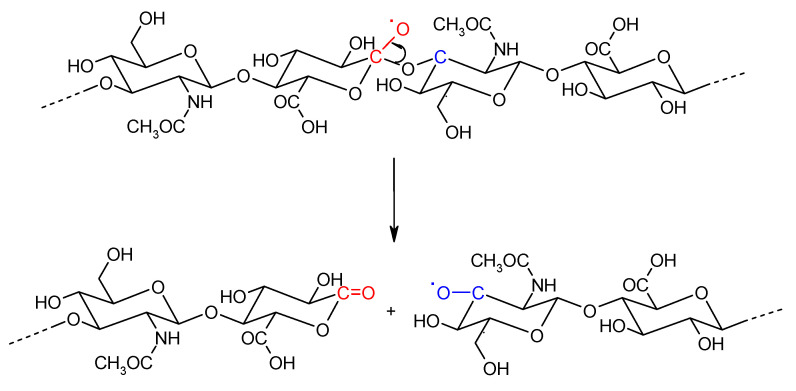
The long chain alkoxy-type macroradical AO^•^ undergoes a transfer of one electron at, e.g., C(1) on the ring of d-glucuronic acid (upper panel). The decay of the native chain alkoxy-type macroradical AO^•^ yields two polymer fragments (lower panel) having reduced molar masses. The newly formed alkoxy-type macroradical of shorter molar size (AO^•^) naturally could act as the initiator of the self-perpetuating free-radical HA degradation.

As is well-known in macromolecular chemistry, a long chain alkoxy-type macroradical freely undergoes the strand scission due to the β-cleavage. Thus the scission at, e.g., C(1) on the ring of d-glucuronic acid, yields polymer fragments, namely a macromolecule bearing a terminal C=O group and a novel alkoxy-type macroradical (cf. Scheme 3). As a rule of such a degradation reaction both fragments have reduced molar mass.

The initiating ^•^OH radical(s) (cf. Scheme 1) can react with the d-glucuronate/d-glucuronic acid and *N*-acetyl-d-glucosamine functional moieties by opening the alkyl rings [52,53,54,55] without breaking the HA chain.

#### 3.1.3. Termination Reaction(s)

The free-radical degradation reactions of the native high-molar-mass hyaluronan belong to self-perpetuating reactions. One of the most effective procedures terminating the self-perpetuating reactions is to scavenge this sequence in the very phase of their initiation. Reactions represented in Scheme 1, subsequently followed by H_2_O_2_ decomposition, can be terminated by a so-called preventive antioxidant, e.g., a substance freely donating an atom of hydrogen, i.e., ^•^H: In such a case the substance, classifiable as HAT—hydrogen atom transferring, terminates the action of the ^•^OH radical and thus it interrupts the generation of A^•^ macroradical. (It should be claimed here that at pH 7.4, more than 99.9% of ascorbic acid (AscH_2_) is present dissociated as AscH^−^. Thus, the antioxidant chemistry of vitamin C is the chemistry of ascorbate, which freely donates a hydrogen atom to an oxidizing radical).

According to the cascade of reactions showed in Scheme 2 and Scheme 3 it is obvious, that to prevent the propagation phase of the free-radical HA degradation one must apply an antioxidant which acts as HAT. Such a property is attributed to the so-called chain-breaking antioxidants. It is comprehensible that the antioxidant acting as chain-breaking should effectively scavenge both the AOO^•^ and AO^•^ radicals. In case of these two *O*-centered radicals, although their standard reduction potential is lower than that for hydroxyl radical ^•^OH, H^+^/H_2_O (+2.31 V) [56], they are still very oxidative—similar to those of an aliphatic peroxyl radical ROO^•^, H^+^/ROOH (≈ +1.0 V) or an aliphatic alkoxyl radical RO^•^, H^+^/ROH (≈ +1.6 V) [57,58,59].

### 3.2. Assessment the Substance Antioxidative Profile by Hyaluronan Plus the Weissberger System

**Figure 3:**
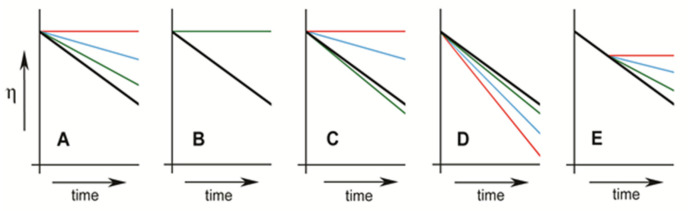
Panels (**A**–**D**) represent the results of assessing the substance functioning as a preventive antioxidant. Panel (**E**) schematically shows the η functional dependence observed when applying a chain-breaking antioxidant.

The idea to use high-molar-mass HA for the evaluation of antioxidants can be traced to the year 1994 [60,61]. At present, the experimental set-up uses the physiologic solution of the HA sample with an average molar mass ≈ 1.5 MDa. The time-dependent changes in dynamic-viscosity values after the application of CuCl_2_ and ascorbic acid are monitored by a rotation viscometry device [62]. In the experimental design when the application of the test substance precedes the addition of ascorbic acid, one examines the preventive antioxidant properties of the substance. On assessing the substance chain-breaking antioxidant properties the addition of substance examined follows the application of ascorbic acid after a properly selected time interval, i.e., during the steady state phase of HA degradation propagation. The panels in Figure 3 schematically illustrate the types of functional time dependencies on dynamic viscosity (η). (Other methods used were infrared spectroscopy [34,35], thermal chemiluminescence [34,35,63] and EPR spectroscopy [35,64], the last one to identify the formation of radicals in selected oxidation systems.) To comment the below represented experimental results it should be claimed that, as repeatedly proved [15,16,17,18,45,51,65,66], the decrease in dynamic viscosity values reflects the decrease in average molar mass of the HA sample with respect to the existing functional dependence η = f (M).

The time dependence of the decrease in the value of dynamic viscosity marked in black on panels A to E of the experimental set-up is as follows: CuCl_2_ and ascorbic acid solution are gradually applied to the HA solution so that the actual concentrations of these components are 2 mg/mL, 1 and 100 μmol/L, respectively. It should also be noted here that the experiments were performed under aerobic conditions, i.e., the concentration of oxygen in aqueous solutions, at standard barometric pressure has been ≈250 µmol/L at 25 °C. When verifying whether a given substance acts as a preventive antioxidant, it was applied in several doses, so that the actual concentration of the substance within the experimental vessel was usually 100 (red), 10 (blue), and 1 (green curve) μmol/L, respectively. The results of antioxidative profile assessment of several substances are given in Table 1.

## 4. Concluding Remarks

We have reported evidence to support our thesis regarding the assessment of the antioxidative efficiency of an endo- or exogenous water soluble substance by exploiting a reaction system comprising high-molar-mass hyaluronan along with cupric cations and ascorbate. The ascorbate level in body fluids of healthy individuals is in the range of 40–140 μmol/L [50]. The total concentrations of copper ions in healthy human beings may reach micromolar levels [96]. Thus during ascorbate (auto)-oxidation in the presence of trace levels of cupric ions as catalysts, direct transformation of O_2_ to H_2_O_2_ happens [7,8,10,11,12,13]. Subsequently, the produced hydrogen peroxide is decomposed by the action of the transition metal counter cuprous cations, which are site-specifically fixed by the HA polyanionic chain and thus ^•^OH radicals interact with HA macromolecules. The generated hydroxyl radicals, and/or mainly the *C*-centered hyaluronan macroradicals, if not scavenged by a proper preventive antioxidant, continue their subsequent self-perpetuating free-radical degradation up to the moment of their reaction with a chain-breaking antioxidant.

## Figures and Tables

**Table 1:** Antioxidative profile assessment of several substances.

Substance	Preventive Action	Chain-Breaking Action	B	C	D	Reference/Note
*N*-Acetylcysteine	Effective	Effective		Effective ^a^		[67,68,69]
Acetylsalicylic acid	Effective	Effective				[70]
Arbutin	Effective	Effective				[71]
Aurothiomalate	Effective	Effective				[72]
Bucillamine	Effective	Effective				[63,73,74,75,76,77]/It is a dithiol.
Captopril	Effective	Effective		Effective		[56,78]
Carnosine	Effective	Effective				[79]
Cemtirestat	Effective	Effective	Effective ^b^			[59]
Cysteamine	Effective	Effective				[80]
l-Cysteine	Effective	Effective		Effective ^a^		[73,81]
Dithiothreitol	Effective	Effective				[77]/It is a dithiol.
Dithioerythritol	Effective	Effective				[77]/It is a dithiol; SH groups’ pK_a_ values = 9.2 and 10.1.
d-Mannitol	Effective	Effective				[61]
Edaravone	Effective	Effective				[82]
Ergothioneine	Effective	Effective				[83]
Isatin	Effective	Effective	Effective ^b^			[59]
l-Glutathione	Effective	Effective		Effective ^a^		[15,35,67,84,85,86]
Glutathione disulfide	Effective	Effective				[67]/It is the oxidized l-glutathione.
Hercynine	Ineffective	Ineffective				[83]
Histidine	Effective	Ineffective				[83]
Homocysteine	Effective	Effective				At neutral pH it is a zwitterion.
Ibuprofen	Effective	Not investigated				[87]/RS-(±)-, R- and S-enantiomers were evaluated.
Levamisole	Ineffective	Ineffective				[78]
Methotrexate	Effective	Effective				[79]
Methylsulfonylmethane	Ineffective	Effective				[88]/Chain-breaker in extremely high dose.
Mn(III)-porphyrins ^c^					Effective ^c^	[89]
Naproxen	Effective	Effective				[70]
d-Penicillamine	Ineffective	Effective				[90,91]/SH group’s pK_a_ value = 10.5
Phosphatidylcholine dihydroquercetin	Ineffective	Ineffective				[92]
Stobadine	Effective					[59,93,94]
Taxifolin	Ineffective	Ineffective				Dihydroquercetin, belongs to a subgroup of flavanols
Tiopronin	Effective	Effective				[56,78]
Vanillin	Effective	Effective				Phenol-type compound
Vinpocetine	Effective	Effective				[61]

^a^ *N*-Acetylcysteine, l-cysteine, and l-glutathione are weak acids, which pK_a_ values of their −SH group equal to 9.52, 10.78, and 9.65, respectively. Due to dissociation of thiol a minute fraction of thiolate anions along with the generated thiyl radicals act both like the reductants of O_2_ molecules yielding superoxide anion radicals, which subsequently participate in reaction with in situ generated H_2_O_2_ molecules according to the reversible Haber-Weiss reaction O_2_^•−^ + H_2_O_2_ ↔HO^•^ + HO^−^ + O_2_ [95]. ^b^ Since both substances, i.e., cemtirestat and isatin strongly intercalate cupric ions, no redox active Cu(II) was available to form ascorbate---Cu(II)/Cu(I)---dioxygen complex even at the lowest substance concentration (1/100). ^c^ Mn(III)meso-tetrakis(*N*-ethylpyridinium-2-yl)porphyrin (MnTE-2-PyP^5+^), in phase II clinical trials, accepts one electron from AscH^−^ and subsequently the Mn(II) center acts as a reductant of O_2_ molecules yielding superoxide anion radical, which subsequently participate in reaction with in situ generated H_2_O_2_ molecules according to the above mentioned reversible Haber-Weiss reaction.

## Data Availability

The data presented in this study is openly available.
